# The nematode *Caenorhabditis elegans* as a tool to predict chemical activity on mammalian development and identify mechanisms influencing toxicological outcome

**DOI:** 10.1038/srep22965

**Published:** 2016-03-18

**Authors:** Philippa H. Harlow, Simon J. Perry, Stephanie Widdison, Shannon Daniels, Eddie Bondo, Clemens Lamberth, Richard A. Currie, Anthony J. Flemming

**Affiliations:** 1 Syngenta Ltd., Jealott’s Hill Research Station, Bracknell, Berkshire, RG42 6EY, UK; 2 General Bioinformatics, Jealott’s Hill Research Station, Bracknell, Berkshire, RG42 6EY, UK; 3 Syngenta, 3054 East Cornwallis Road, Research Triangle Park, NC 27709-2257, USA; 4 Syngenta Crop Protection AG, Chemical Research, Schaffhauserstrasse 101, 4332 Stein, Switzerland

## Abstract

To determine whether a *C. elegans* bioassay could predict mammalian developmental activity, we selected diverse compounds known and known not to elicit such activity and measured their effect on *C. elegans* egg viability. 89% of compounds that reduced *C. elegans* egg viability also had mammalian developmental activity. Conversely only 25% of compounds found not to reduce egg viability in *C. elegans* were also inactive in mammals. We conclude that the *C. elegans* egg viability assay is an accurate positive predictor, but an inaccurate negative predictor, of mammalian developmental activity. We then evaluated *C. elegans* as a tool to identify mechanisms affecting toxicological outcomes among related compounds. The difference in developmental activity of structurally related fungicides in *C. elegans* correlated with their rate of metabolism. Knockdown of the cytochrome P450s *cyp-35A3* and *cyp-35A4* increased the toxicity to *C. elegans* of the least developmentally active compounds to the level of the most developmentally active. This indicated that these P450s were involved in the greater rate of metabolism of the less toxic of these compounds. We conclude that *C. elegans* based approaches can predict mammalian developmental activity and can yield plausible hypotheses for factors affecting the biological potency of compounds in mammals.

Ensuring the safety to humans of the chemicals they may be exposed to is of critical importance to chemical companies, regulatory authorities and the public. It is in the interest of chemical companies researching new active ingredients (AI) to identify adverse toxicological outcomes as soon as possible and avoid wasted investment in unsafe or unregisterable chemical products. One approach is to test new AI earlier in research programmes using the standard, guideline, mammalian toxicological tests required by regulators to determine toxicological outcomes. However, this implies a substantial increase in the number of mammals used which is undesirable for ethical and economic reasons. Therefore much research has investigated alternative experimental systems that have fewer of these concerns including *in silico* modelling^1^, cell-based systems^2^, vertebrate systems of reduced concern e.g. Zebrafish^3^ as well as invertebrate model systems^4^,^5^. Developmental toxicity, where a chemical adversely affects the biological processes of development from egg to adult, is of concern to the agrochemical industry. Developmental biology has been extensively studied in invertebrate model systems making them obvious candidates for the study of developmental toxicity.

The nematode *Caenorhabditis elegans* is exceptionally well studied and many researchers have used it as a model for different forms of toxicity^5^,^6^,^7^. Its small size, short life cycle and ease of maintenance and culturing make it a viable model for high-throughput screening, while the array of genetic tools available for use with it enable further investigations into the causes of toxicity. Several studies have used *C. elegans* as a model for: the neurotoxicity of xenobiotics^8^, neurodegeneration^9^, genotoxicity^10^, and germline toxicity^5^ and all found relevance of the model to man^11^. For example, the toxicity of a group of organophosphates was shown to correlate between *C. elegans* and mammals^12^. *C. elegans* has also been used to investigate the basis of the toxicity of ethanol^13^, volatile anaesthetics^14^ and other drugs^15^,^16^,^17^.

*C. elegans* development is fully described^18^,^19^ and the underlying genetic mechanisms controlling development are well understood and often conserved with those found in mammals^20^. Embryogenesis, from fertilization to egg hatching takes approximately 13–14 h at 20 °C and produces 671 cells (113 of which die by apoptosis) forming the L1 *C. elegans* larva^19^. Numerous developmental processes occur, leading to cell fate specification, tissue formation and morphogenesis. Scoring egg viability by counting the number of eggs that hatch as a proportion of those laid is therefore a convenient and quantitative measure of the success of these developmental processes. Chemicals or other exogenous factors affecting developmental biology are likely to affect egg viability.

Chemical toxicity results from a xenobiotic molecule adversely affecting a process or function upon which a toxicological outcome is contingent. Typically this will be the consequence of a biochemical interaction between the small molecule and an endogenous protein or proteins involved in this process or function. Therefore a major determinant of toxicology is the potency of this biochemical interaction. But this is not the only determinant, also important is the distribution and abundance of the small molecule in the organism and its consequent availability with respect to the target protein(s) driving the toxicological outcomes. The importance of absorption, distribution, metabolism and excretion on the interaction between small molecules and organisms has long been recognised^21^. Of these, metabolism has been extensively researched, not least because of its importance to the efficacy of pharmaceuticals. An important class of metabolising enzyme is the Cytochrome P450 enzymes which are a superfamily of NADPH-dependent monooxygenases that catalyse the Phase I metabolism of xenobiotics such as pesticides^22^. The *C. elegans* genome contains 77 intact cytochrome P450 genes^23^. Differences in toxicity among compounds could be caused by differences in affinity for a single P450 that metabolises both compounds, metabolism of compounds by more than one P450 or changes in gene expression of P450 gene(s) responsible for metabolism of one or both compounds.

In this study we assessed the utility of *C. elegans* for toxicological investigations, and in particular for generating hypotheses relevant to human safety. We did this in two ways. First, we screened diverse pesticide chemistry, including compounds with mammalian developmental activity in the ToxRef database^24^, and measured their developmental activity in *C. elegans*. This allowed us to estimate the correlation of chemically-induced developmental activity between nematodes and mammals and therefore the predictive power of one system on the other. Definitions of toxicity (including developmental toxicity), which are considered in the registration and labelling of commercial products, vary among jurisdictions and change over time. Even fundamental concepts such as the relative importance of ‘risk’ and ‘hazard’ are debated in this context^25^ and can cause controversy^26^. This makes it hard to identify a suitable set of universally accepted, developmentally toxic standards which is needed to evaluate predictive approaches such as the one we describe. To overcome this, we chose to work on compounds that were reported simply to have more potent biological activity in developing, mammalian embryos than in adults, from the ToxRef database^24^. While such compounds cannot be considered developmentally toxic on the basis of these data alone, they have activity on the developing, early life stages of mammals, which could result in developmental toxicity, We reasoned that a tool that predicted developmental activity and so the possibility of developmental toxicity, could be useful in prioritizing and directing subsequent toxicological investigation. Whether such a compound was ultimately classified as developmentally toxic would depend on these subsequent studies and on the regulatory definitions of toxicity in relevant jurisdictions. In the second part of our study, we focussed on a closely related series of proprietary pyridazine and imidazole fungicides recently dropped from Syngenta’s research portfolio because developmental toxicity was observed with some examples of the series. We asked whether *C. elegans* could identify factors underlying the toxicology of the series and suggest approaches to, in principle, redesign molecules with improved toxicological profiles.

## Results

### The egg viability assay in *C. elegans* as a screen for developmental toxicity in mammals

We assessed the utility of *C. elegans* as a system used to screen for compounds with developmental activity. We selected 72 pesticide compounds from the ToxRef database as a test set^24^,^27^. This database contains summarised results from studies submitted to the US Environmental Protection Agency as part of the registration of pesticides. The initial build contained 1318 records of prenatal developmental studies, conducted on rats or rabbits. Endpoints recorded included maternal effects such as body weight gain, food and water consumption, fertility and pregnancy, as well as foetal effects such as foetal weight reduction, skeletal variations, malformations and other pathologies^24^. The database provides a lowest effect level (LEL) for both maternal toxicity and developmental effects (a measure of toxicological potency to the embryo) in mammals.

If a compound had a lowest effect level for developmental effects at a lower concentration than for adult toxicity it was considered to be developmentally active because it had effects on embryo development in the absence of effects on the mother. We considered effects occurring at doses when the mother was sick might be indirect maternal effects rather than developmental effects *per se*. Using these criteria 57 of the selected compounds were developmentally active; the remaining fifteen compounds were negative controls. A full list of compounds is provided as supplementary material (Supplementary Table 1). The compounds represent diverse structures and mechanisms of action including insecticides, fungicides and herbicides.

These compounds were then tested to determine if they affected *C. elegans* egg viability. L4 *C. elegans* were exposed to compounds for 48 h and allowed to lay eggs. The adults were then removed and the number of unhatched eggs still remaining after 24 h recorded. If the number of unhatched eggs significantly exceeded control levels the compound was considered to reduce egg viability (and therefore to be developmentally active) in *C. elegans*. Control levels were a mean number of unhatched eggs of 1.41 per well with a standard deviation of 1.72. Differences from control were measured by t-test (p < 0.001).

Nineteen compounds reduced egg viability in *C. elegans*, of which seventeen were from the group defined as developmentally active in mammals (Table 1). The positive predictivity of this assay is therefore 89%. In other words 89% of compounds found to be developmentally toxic in our assay in *C. elegans* are also developmentally active in mammals. Analysis of the complete ToxRef database shows the percentage of compounds found to be developmentally active in mammals ~18% 24. Based on this finding, our assay improves this prediction markedly. If a compound is active in our assay it is ~5 fold (89% versus 18%) more likely to be developmentally active in mammals compared to this “baseline” expectation.

However only 25% of compounds found to not to affect egg viability in *C. elegans* are also not developmentally active in mammals. The negative predictivity of the assay is therefore low, relative to the positive predictivity. Based on these data, we conclude that a positive result in the assay is likely to accurately predict mammalian developmental activity, while a negative result in this assay only weakly predicts that a compound will not be developmentally active in mammals. Therefore the majority of mammalian developmentally active compounds will be inactive in this assay; however a compound that is active in the assay is likely to be developmentally active in mammals.

### The egg viability assay in *C. elegans* as a tool for investigating differential toxicity across a related series of compounds

Commercial synthetic chemistry research typically produces a series of analogue compounds all structurally related to an initial lead compound. The purpose of this synthetic effort is to understand the impact of structural modifications on the properties of the chemical. This can then enable the rational design of molecules with desirable properties, which may include an improved toxicological profile. Therefore having demonstrated the ability of a *C. elegans* egg viability assay to identify mammalian developmentally active compounds within a diverse chemical collection, we now looked at the utility of this assay within a series of closely related compounds. The compounds we chose are a series of proprietary pyridazine and imidazole fungicides which disrupt microtubule dynamics^28^,^29^.

**Figure 1:**
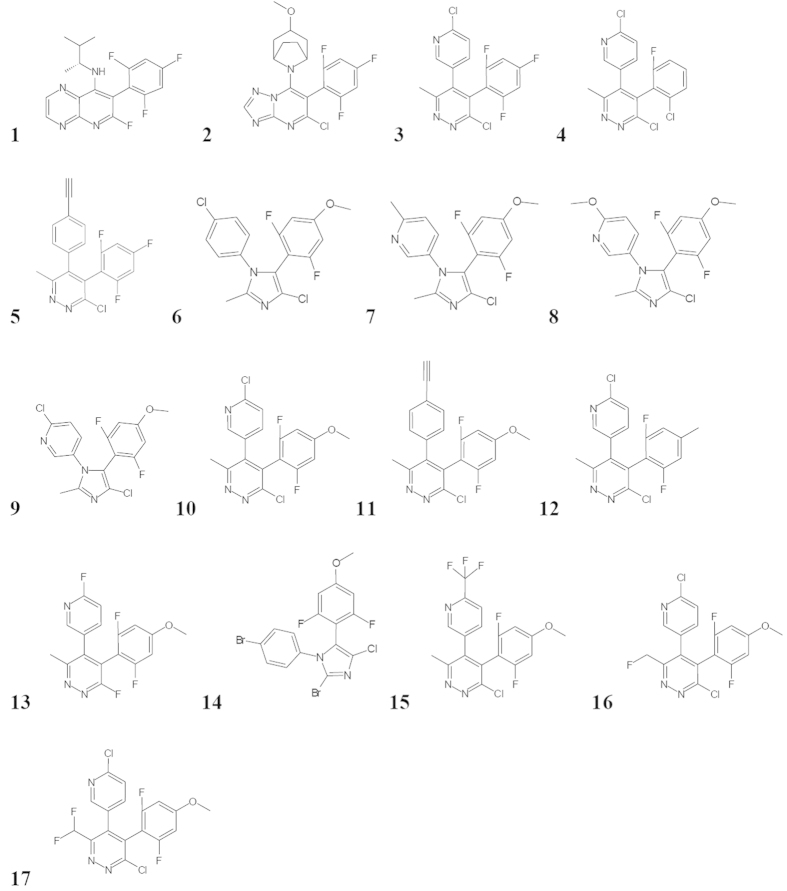
A series of imidazole, pyridazine, pyridopyrazine and triazolopyrimidine fungicides that act by disrupting microtubule dynamics^28^,^29^.

Seventeen of these compounds were tested in the egg viability assay (Fig. 1). These compounds were selected by Syngenta as having commercial potential and therefore suitable for further research. Compounds **2**, **3**, **10** and **15** have been shown to cause teratogenicity in rats. Compounds **5** and **6** have not shown clear developmental toxicity in the same preliminary tests (Supplementary Table 2). There is no mammalian data for the remaining compounds. All the compounds were biologically active in *C. elegans*. We considered that the mechanism of action in *C. elegans* was likely to be related to that in fungi i.e. the disruption of microtubule function. Fungicidal chemicals acting in this way have previously been shown to also be active on *C. elegans*^30^.

**Figure 2:**
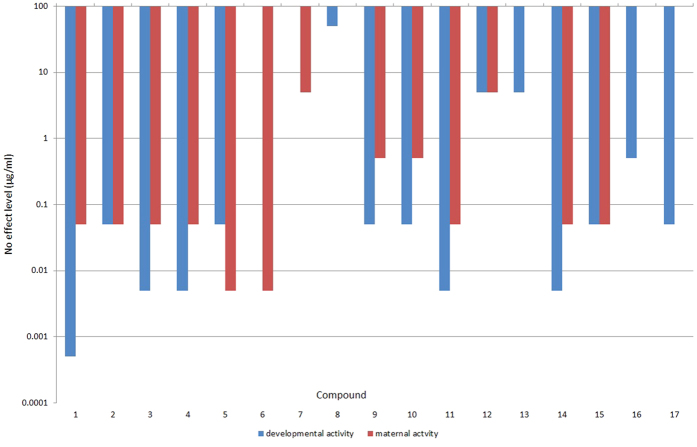
Different compounds in this series affect egg hatching to different extents. The maternal activity and developmental activity in *C. elegans* of a series of compounds **1-17** (See Fig. 1) tested in the egg viability assay. The blue columns show the No Effect Level (NOEL) for developmental activity i.e. the highest concentration at which no significant (p < 0.001) effect on egg viability was observed. The red columns show the NOEL for maternal activity: the highest concentration at which no adult toxicity was observed. Where no column is present no significant activity was observed at any dose.

We found that, as in mammals, examples from the chemical series induced developmental toxicity (measured as egg viability) in *C. elegans* though the exact pattern of toxicity for different analogues varied between species (Fig. 2). In *C. elegans*, most showed a similar (within one order of magnitude) No Effect Level (NOEL) for both maternal and developmental toxicity. However compounds **6** and **7** showed no developmental toxicity but were maternally lethal. Conversely compounds **8**, **13**, **16** & **17** showed no maternal lethality but were developmentally toxic. Additionally compound **1** had a developmental NOEL two orders of magnitude higher than its maternal NOEL. This variation in induced developmental toxicity presented an opportunity to investigate the mechanism(s) underlying the developmental toxicity of this series of compounds in *C. elegans*.

### Differences in metabolic stability in *C. elegans* between related compounds

We selected six compounds for further study based on their differing toxicological effects on *C. elegans*. These fell into three groups. Group A comprised compound **3** and compound **4** which are pyridazine compounds that showed high levels of both maternal toxicity and egg toxicity. Group B comprised compound **6** and compound **7** which are imidazole compounds that showed low egg toxicity and high/medium maternal toxicity. Group C comprised compound **16** and compound **17** which are also pyridazine compounds and these showed low maternal toxicity and medium egg toxicity.

**Figure 3:**
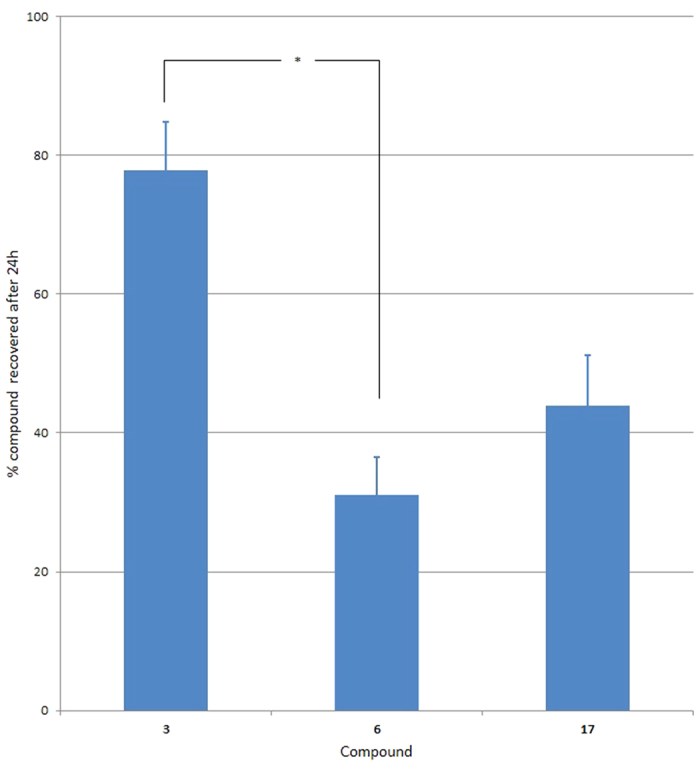
*C. elegans* metabolism assay. Data obtained by LC-MS analysis of samples containing compounds **3**, **6**, and **17**. Compound **6** is metabolised to a greater extent in 24 h than Compound **3**. The data are expressed as a percentage of recovered compound at time 24 h vs. time 0 h. The columns show the average ± s.e. of n = 3. *indicates p < 0.05 from a t-test.

A possible cause of the toxicological differences between compounds could result from variations in bioavailability, e.g. differential metabolism, affecting the exposure of either the egg or adult to the compound. To address this, we measured the rate of metabolism of one compound from each of the three groups (Groups A to C, Fig. 1 compounds **3**, **6**, **17**, respectively) by investigating its metabolic stability in nematodes over a 24 h period (Fig. 3). The data are expressed as a percentage of recovered compound at time 24 h vs. time 0 h. The *C. elegans* metabolism assay and LC-MS analysis of extracts is described in detail in the Methods section. Overall, compound **6** had the greatest loss in 24 h; compound **3** had the least. These were significantly different (p = 0.013) levels of metabolism. Compound **17** showed an intermediate level of metabolism, closest to compound **6**. So the compound (**6**) showing the highest developmental toxicity NOEL (i.e. the least developmentally toxic) is the least metabolically stable and the compound (**3**) with the lowest developmental toxicity NOEL (the most developmentally toxic) shows the lowest rate of metabolism after 24 h. Compound **17** is intermediate for both measures. We conclude that reduced developmental toxicity among the test compounds is associated with increased rate of loss of the compound through metabolism. We then investigated the mechanism of increased rate of metabolism.

### The cytochrome P450 genes *cyp-35A2-5* and *cyp-35C1* are upregulated by compound 6 and compound 17 but not compound 3

Xenobiotics are known to induce the expression of metabolic enzymes^31^,^32^. Therefore the differential metabolism we observed among analogues might result either from their intrinsic susceptibility to metabolism or from their ability to induce metabolic gene expression. We performed a microarray to establish which genes were altered in expression in response to a 48 h exposure to the six, selected compounds.

**Figure 4:**
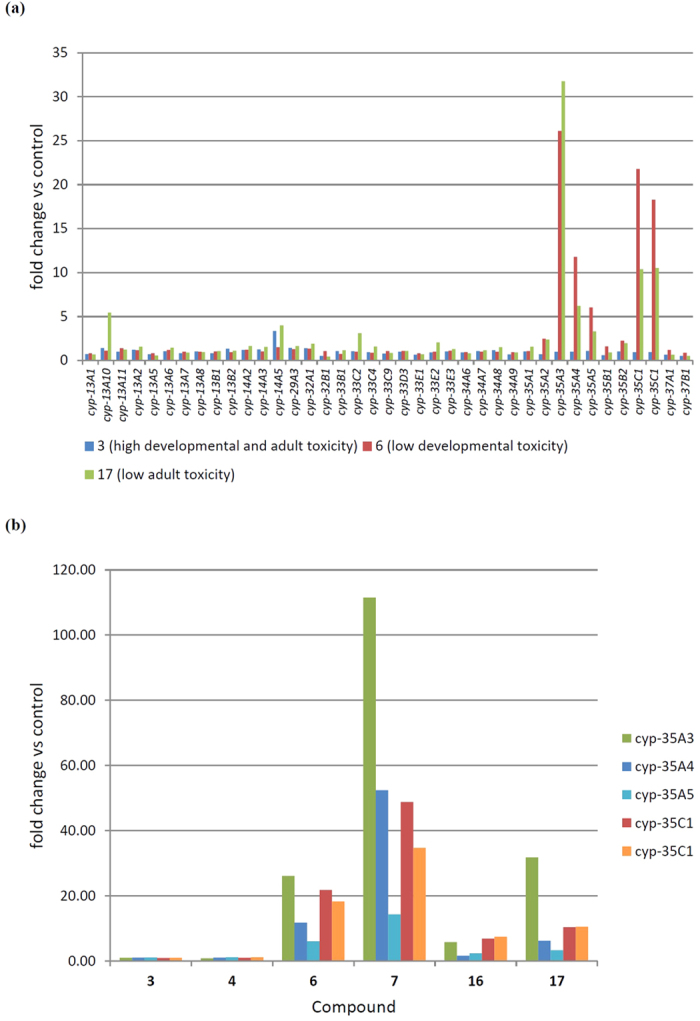
Compound 6 and compound 17 both cause upregulation of *cyp-35A3, cyp-35A4, cyp-35A5* and *cyp-35C1* whereas compound 3 does not. (**a**) Fold upregulation of cytochrome P450 genes in response to three compounds. Only genes showing detectable expression are shown (**b**) Fold upregulation of *cyp-35A3, cyp-35A4, cyp-35A5* and *cyp-35C1* in all six compounds. Two oligonucleotides in the microarray targeted the *cyp-35C1* gene, both showed a similar pattern of induction. See Supplementary Table 3 for full fold change data of all differently expressed cytochrome P450 genes.

We find that the cytochrome P450s *cyp-35A2-5* and *cyp-35C1* are upregulated by some, but not all of our test compounds (Fig. 4, Supplementary Table 3). They were upregulated to the greatest extent in compound **7** and thereafter in the order compound **6**> compound **17**> compound **16**, except that only *cyp-35A3* and *cyp-35C1* showed significant upregulation in response to compound **16** and *cyp-35A3* showed slightly greater upregulation in compound **17** than compound **6.** None of these genes were upregulated at all in compound **3** and compound **4**. This mirrors the *C. elegans* developmental toxicity data for these compounds. Therefore, we asked whether these metabolic genes were involved in the faster metabolism of the less developmentally toxic of these compounds.

### RNAi knockdown of *cyp-35A3* and *cyp-35A4* together causes *C. elegans* eggs to fail to hatch after exposure to compound 6 or compound 7

We wanted to determine whether cytochrome P450s were involved in the observed differences in developmental toxicity of these compounds and if so which ones. We targeted 58 cytochrome P450 encoding genes with RNAi and determined the effect of knockdown on the developmental toxicity of compounds **6** and **7** (Table 2). To keep the experiment manageable we knocked down the expression of genes in groups of up to 3 (as previously described^33^), and scored the subsequent effects on egg hatching following exposure to the test compounds (see Methods). RNAi knockdown of most of the cytochrome P450s tested had no effect on the developmental toxicity caused by either compound. Simultaneous knock down of *cyp-35A2*, *cyp-35A5* and *cyp-35C1* resulted in a small, non-significant (**6**, 0.5 μg/ml, p = 0.114, **7**, 50 μg/ml p = 0.203) increase in developmental toxicity which we did not investigate further. Only simultaneous knockdown of *cyp-35A3* and *cyp-35A4* showed a significant increase in developmental toxicity (**6**, 0.5 μg/ml, p = 0.004, **7**, 50 μg/ml p = 2.53 × 10^−6^). 79% of eggs exposed to 50 μg/ml of compound **7** and 89% of eggs on 0.5 μg/ml of compound **6** did not hatch. This was compared to 9% and 6% respectively in controls in which the compound was present in the absence of the RNAi treatment. In controls in which the RNAi treatment was present in the absence of the compound the rate was 1%.

**Figure 5:**
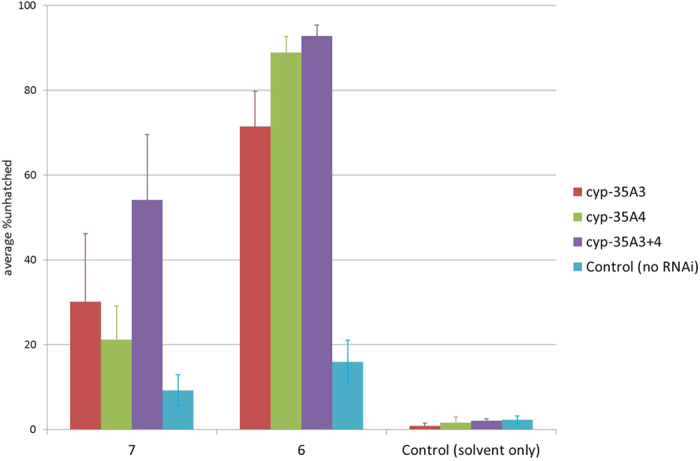
c*yp-35A3* and *cyp-35A4 RNAi* worms are more sensitive to the developmentally toxic effects of 7 and 6. We used 0.5 μg/ml and 50 μg/ml doses of compounds **6 & 7** respectively. These doses induce mild maternal toxicity in *C. elegans* (N2) See Fig. 2. *C. elegans* developmental toxicity is greatly increased in (*cyp-35A3 RNAi* and *cyp-35A3 RNAi* together) compared to N2. *C. elegans* developmental toxicity is also increased in response to *cyp-35A3 RNAi* and *cyp-35A4 RNAi* separately but not to as great an extent. The columns show mean ± s.e. of n = 4.

Separate knockdown of each gene individually also resulted in reduced developmental toxicity suggesting that either it is influenced by both enzymes or that RNAi targeted to one cytochrome P450 has effects on another (Fig. 5). Interestingly, we also included compound **4** in these experiments and found no evidence that these enzymes affected the toxicological effects of this compound (Supplementary Fig. 1). We conclude that the expression of *cyp-35A3* and/or *cyp-35A4* is a major determinant of the developmental toxicity of compounds **6** and **7** in *C. elegans*.

## Discussion

We evaluated a *C. elegans* based approach for toxicological research. We first asked whether *C. elegans* could be used as an alert for the potential of a research compound to cause developmental effects in mammals. Second, we asked whether *C. elegans* could be used to reveal mechanisms driving the toxicological effects of compounds, mechanisms that, if understood, might enable mitigation of the effects.

For the first component, we find that the positive predictive power of the *C. elegans* egg viability assay we employed is surprisingly high: 89% of compounds found to be developmentally active in *C. elegans* by this measure are also developmentally active in mammals (as noted previously whether a compound would be classified as developmentally toxic would depend on subsequent experiments and regulatory oversight). A strength of our assay is that it includes both the mother (a *C. elegans* hermaphrodite) and the developing embryo. Once laid the eggshell will likely limit chemical ingress to the embryo^34^, the assay therefore models embryonic exposure via maternal exposure, as in mammalian tests. Furthermore, the assay records the toxicity to the egg relative to the toxicity to the mother, we suspect this relative toxicity measure controls for the effects of scale that could otherwise confound correlations between *C. elegans* and mammalian effects.

However the negative predictivity of the assay was low: 25% of compounds found not to be toxic to *C. elegans* were also not developmentally active in mammals. We record only one endpoint, egg viability and it is possible that other *C. elegans* assays, looking for additional developmental perturbations would identify compounds missed by the assay reported here; such perturbations could include those observed as developmental phenotypes by geneticists^35^. However accuracy will always be limited by intrinsic differences between mammals and nematodes, while *C. elegans* shares many developmental processes with mammals, it does not share them all. Processes associated with the formation of structures not present in *C. elegans*, the skeleton for example, can be only incompletely represented in *C. elegans* at best and this may underly the weak negative predictivity we observed.

An example of a chemical research project dropped due to adverse toxicological outcomes is the one that produced the pyridazine and imidazole fungicides examined in the second component of our study. Here we investigated the potential for *C. elegans* to provide mechanistic insights into toxicology that could, in principle, be exploited to design less toxic compounds. We show that the expression of the genes *cyp-35A3* and/or *cyp-35A4* are required for the reduced toxicity of compounds **6** and **7** while having no impact on the biological activity of other compounds from the chemical series. Biological differences between close chemical analogues are hard to predict and are valuable in revealing subtle effects of structure on biological activity within closely related compounds.

Several studies have investigated the upregulation of P450 enzymes in *C. elegans* in response to various xenobiotic compounds^36^,^37^,^38^,^39^,^40^. The CYP-35 genes in particular have been shown to be strongly inducible^38^. Fewer studies have tried to identify the enzyme that metabolises a given xenobiotic. In one example, the enzymes *cyp-14A* and *cyp-34A6* were identified as the major contributors to the metabolism of PCB52 in *C. elegans* by directly measuring the formation of hydroxylated metabolites whose production required the expression of these enzymes^33^.

Genetic interactions between cytochrome P450 encoding genes and xenobiotic compounds such as those we and others have observed, may arise for different reasons. Firstly compounds may act directly on cytochrome P450s to deliver their toxicological outcome i.e. they may themselves be the target of the compound. Several molecules are known to inhibit cytochrome P450s, including piperonyl butoxide (PBO)^41^. Second, cytochrome P450s may act to metabolise the compound to a more or less biologically active metabolite and so modulate toxicological outcomes of the original compound. For example the toxicity of the organophosphate fenitrothion and its actions on its target, acetylcholine esterase, was shown to be reduced by knockout of *cyp-35A2*. This was taken to indicate that this P450 was involved in its biotransformation to the active form^42^. Thirdly, the effect may be indirect: cytochrome P450s have endogenous functions including the metabolism of fat into which lipophilic compounds may partition and so be sequestered away from their target proteins. Under these circumstances changes in fat metabolism might therefore indirectly affect toxicity by altering the sequestration of toxic compounds. Knockout mutants of genes of the *cyp-35A* subfamily have been shown to have reduced fat storage^43^,^44^, which has been implicated in their role in the toxicity of PCB52^33^. However they have also been shown to be involved in xenobiotic metabolism^42^.

**Figure 6:**
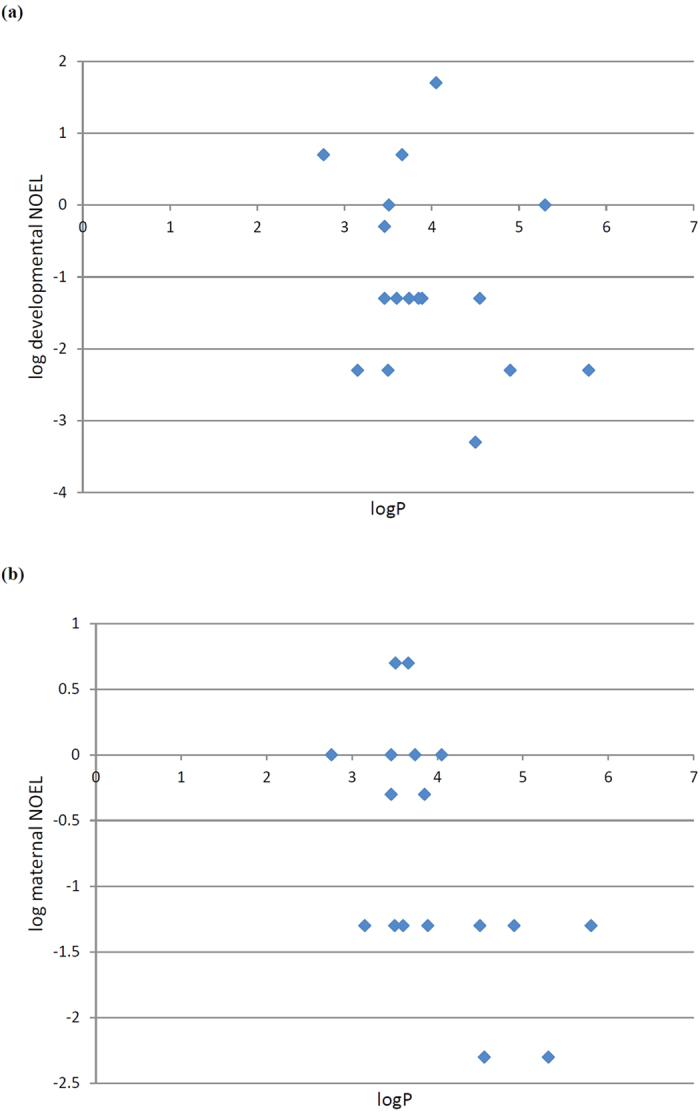
Correlation of maternal and developmental toxicity with lipophilicity. There was no correlation between the logP of the compounds (a measure of lipophilicity) and their (**a**) developmental NOEL in *C. elegans* or (**b**) maternal NOEL.

We cannot formally distinguish these possibilities on the basis of our experiments. That said our knowledge of the mechanism of action of this compound series does not suggest they are toxic because of direct effects on cytochrome P450 enzymes (they are microtubule disruptors). Furthermore we find no correlation between the logP (a measure of lipophilicity) of the compounds and toxicological potency which does not suggest that partition into fat stores explains the toxicological differences among these compounds in *C. elegans* (Fig. 6). Rather, we suggest that *cyp-35A3* and/or *cyp-35A4* are enzymes involved in the metabolism of compounds **6** and **7**, and that the reduced toxicity of these compounds compared to the closely related compounds **3** and **4** is due to their reduced bioavailability as a result of their greater rate of metabolism by these enzymes.

The cytochrome P450s *cyp-35A2-5* and *cyp-35C1* were upregulated in response to compound **6** and compound **7** which showed the lowest levels of developmental toxicity in *C. elegans*. This upregulation was clearest in *cyp-35A3, cyp-35A4* and *cyp-35C1*. It is likely that the differential bioavailability of these compounds might be due to their upregulation of *cyp-35A3* and/ or *cyp-35A4* which therefore metabolises them faster. However *cyp-35C1* which is massively upregulated in response to these compounds does not appear to play a role in their toxicity. This reflects what was found by Schäfer *et al*. in their study of the metabolism of PCB52. They found that the enzymes *cyp-14A* and *cyp-34A6* metabolised PCB52^33^. In earlier studies PCB52 had induced the expression of many different P450s including *cyp-14A3, cyp-34A10, cyp-35A* and *cyp-35C1*^36^,^37^,^38^ but no induction of expression of *cyp-34A6* has been reported. Therefore in this case the induction of cytochrome P450 genes including *cyp-35C1* was not indicative of them being involved in the metabolism of the compound. However *cyp-14A3* was both induced by, and involved in the metabolism of PCB52. In addition *cyp-35A2* has been shown to be both induced by, and involved in the toxicity of, the compound fenitrothion^42^.

In mammalian systems, where more is known about the responses of cytochrome P450 to xenobiotics, there is no automatic assumption that an inducer or inhibitor of a P450 will be metabolised by it. This is certainly sometimes the case, for instance chronic exposure to ethanol will upregulate CYP2E1 which is the enzyme that metabolises ethanol^45^. However several P450 inducers or inhibitors such as paroxetine induce/inhibit more P450s than just the one they are metabolised by^46^. Together with the observations described here, this underlines the importance of combining genetics with analytical chemistry, and with measures of biological activity in the whole organism, to be sure of the functional contributions of metabolic enzymes.

The industry-wide impact of unintended toxicological outcomes during agrochemical research and development has not been calculated, but is certainly significant. In the pharmaceutical industry, non-clinical toxicology (which includes adverse findings in animal tests) is estimated as the most frequent (40%) cause of attrition in the drug development pipeline^47^. Therefore, a substantial improvement in pipeline efficiency would be achieved if compounds likely to fail through non-clinical toxicology were identified earlier and either dropped or redesigned. One way to achieve this would be to perform toxicity testing earlier in the pipeline, but, using conventional approaches, this would inevitably lead to increased animal testing and is therefore unacceptable. Only the use of predictive tools, such as those we describe here, offer practical means to achieve earlier assessment of toxicology. Many groups are currently investigating this possibility using various approaches such as *in silico* modelling^1^ and cell-based systems^2^, as well as using other model organisms such as the slime mould *Dictyostelium discoideum* and the zebrafish *Danio rerio*^3^,^48^.

Once the risk of adverse toxicological outcomes in mammals has been identified, hypotheses on the factors determining these outcomes may ultimately help to avert the risk through chemical design. We show that metabolism and the actions of particular cytochrome P450 enzymes are determinants of *C. elegans* developmental toxicity which is itself predictive for mammalian developmental activity. An appeal of performing such studies in whole organisms is that mechanisms can be directly linked to toxicological endpoins in the study organism: we show that the function of P450s in *C. elegans* is associated with egg viability when exposed to particular compounds. Whether metabolism by orthologous enzymes affects the developmental toxicity caused by these compounds in mammals, is beyond the scope of this study and is therefore not known. But, more generally, an effect of cytochrome P450 mediated metabolism on the biological potency of compounds in mammals has been frequently observed^49^ and we suggest that it is at least plausible that our findings in *C. elegans* would be relevant to mammals. If so, then designing chemical analogues with the metabolic properties of compounds **6** and **7** would reduce the risk of mammalian developmental toxicity.

In summary, we propose that the egg viability assay in *C. elegans* we describe can be a valuable component of predictive approaches for mammalian developmental toxicity and that *C. elegans* can be used to develop mechanistic hypotheses about effects, including toxicological endpoints, relevant to mammals.

## Methods

### Egg viability assay

We have defined developmental toxicity in *C. elegans* as a reduction in egg viability. The egg viability assay was performed in 24 well plates containing 0.5 ml NGM agar per plate and seeded with 25 μl *E. coli* OP50. AI was added to the plates in 30 μl of solvent (10%DMSO, 50%IPA and 40% H_2_O) per well. Initial tests were conducted at final concentrations of 500, 50, 5 and 0 μg/ml (this last was the vehicle control). However if a compound was inactive at all concentrations it was repeated at 1000 μg/ml and if it was lethal at all concentrations it was repeated at lower concentrations (by ten-fold dilution) until no effect was seen.

Five L4 worms per well were added to the plate and left for 48 h at 20 °C. At this point they were scored for adult toxicity (alive/sick/dead) and removed. The eggs that had been laid were left for 24 h at 20 °C to hatch. The number of eggs per well that remained unhatched was recorded.

By coincidence the mean number of unhatched eggs found in control (solvent only) wells was 1.41, with a standard deviation of 1.72, in both the egg viability assay on the ToxRef compounds (n = 124) and the egg viability assay on the fungicide compounds (n = 52) separately. Compounds were considered to be developmentally active if the mean number of unhatched eggs per well found in response to a given dose of the compound was significantly greater than control (measured by two-tailed Student’s t-test, p < 0.001) (n = 2−6).

For the egg viability assay on the fungicide compounds the no effect levels (NOEL) of the compounds were calculated. The no effect level for developmental toxicity was the highest concentration tested at which the mean number of unhatched eggs was not significantly greater than control (measured by t-test, p < 0.001). The no effect level for maternal toxicity was the highest concentration tested at which the adults appeared indistinguishable from controls. Initial tests were performed on a wide range of doses and then repeated at relevant doses close to the NOEL. Therefore while over the whole dose range n = 2−6 for the doses closest to the NOEL n = 4−6.

The egg viability assay was altered for the RNAi screen to allow the extent of toxicity at a single dose under different conditions to be compared. Two concentrations of each compound were used. These were; compound **6** 0.5 and 0.05 μg/ml, and compound **7** 50 and 5 μg/ml. L4 were left for 24 h to lay eggs. After 24 h they were removed and the number of eggs laid was counted. These eggs were then left for 24 h to hatch and the number of unhatched eggs was counted. The results were expressed as the percentage of the eggs laid that did not hatch.

### *C. elegans* metabolism assay

*C. elegans* were cultivated in liquid bulk culture for one week^50^. Nematodes were treated with imidacloprid, at a rate of 500 μg/ml, 48 h prior to treatment, to induce cytochrome P450 expression. Healthy nematodes were separated by sucrose floatation, washed with 0.1 M cold NaCl at least three times and resuspended in M9. Following centrifugation at 1500 rpm, the supernatant was removed and the nematodes were used in the metabolism assay immediately.

The *C. elegans* metabolism assay involved nematodes (100 μl of bulk culture pellet), added to 24 well plates containing the AI (5 μL in DMSO to make a final concentration of 5 μg/ml) and M9 (to a final volume of 500 μL). Separate control plates contained an additional 100 μl M9 instead of nematodes. Further control plates were prepared in the same way but without treatment with the AI (this was a vehicle control). The plates were shaken continuously for 24 h at 20 °C. The metabolism assay was stopped at 0 h or 24 h by the addition of 500 μl acetonitrile to each well, and the plates were frozen.

Nematode lysis was conducted by the following method. The 24 well plates were defrosted at room temperature and the contents of each well were pipetted into an eppendorf, which was frozen in liquid nitrogen and defrosted immediately in the sonicator bath. The samples were homogenised by 2 × 20s cycles with a FastPrep FP120 (Bio101/Savant) then centrifuged at 10,000 rpm for 15 mins to separate solid debris. The supernatant from each sample was transferred into an HPLC vial and all extracts were analysed by LC-MS. If the extracts could not be analysed immediately they were stored at 4 °C overnight and allowed to warm to room temperature prior to LC-MS analysis. The control samples, without AI, were pooled. The contents of the two plates of control samples, containing *C. elegans*, or saline alone were used to make blank controls and calibration curves.

### Liquid Chromatography – Mass Spectrometry (LC-MS) Analysis

Reversed-phase UPLC analysis was carried out using a ACQUITY UPLC system (Waters, Elstree, UK) and a ACQUITY UPLC BEH C18 column (1.7 μm; 50 × 2.1 mm; Waters, Elstree, UK) with a mobile phase mixture of 0.2% formic acid (A) and acetonitrile (B). During the complete 6-min chromatographic cycle time the linear gradient program was as follows: initial 5% B held for 0.5 min, 5% B increasing to 95% by 4.5 min, 95% B held between 4.5 and 4.9 min, then reduced to 5% B in 0.1 min and 5% B between 5.0 and 6.0 min. The injection volume was 5 μL. A constant flow rate and temperature of 0.7 ml/min and 40  °C, respectively, were maintained throughout the run and the mobile phase was split before reaching the electrospray ionisation mass spectrometry interface. Mass spectrometric analysis was performed with a Micromass ZQ (Waters, Elstree, UK) spectrometer. The instrument was operated in positive ion mode employing single-ion recording (SIR) mode at the molecular ion mass [M + H]+ of each compound; inter-scan delay 0.1s and dwell 0.05s. Matrix-matched standard solutions of each AI were analysed alongside the metabolism assay extracts and data processing was performed using MassLynx (Waters). The data are expressed as percent of recovered compound vs. time 0. The compounds tested were **3**, **6** and **17**.

### Microarrays

Mixed stage *C. elegans* were exposed to AI on plates for 48 h. The compounds were added to the plates in the solvent mixture 10% DMSO 50% IPA 40% H_2_O to the following final concentrations: compound **3** 0.5 μg/ml, compound **4** 0.5 μg/ml, compound **6** 0.5 μg/ml, compound **7** 50 μg/ml, compound **16** 5 μg/ml and compound **17** 10 μg/ml. These concentrations were chosen as being ones in which the compounds caused effects on egg hatching but not adult lethality. The final concentration of solvent was 1%. Three biological replicates were used per compound. The worms were washed and total RNA was extracted using Trizol.

500 ng total RNA per sample was used to create labeled aRNA target using the Affymetrix 3′ IVT Express Kit. 12.5 μg of each of the resulting aRNAs was fragmented and hybridized to the Affymetrix GeneChip^®^ *C. elegans* Genome Array, and then washed and stained using the GeneChip^®^ Hybridization, Wash, and Stain Kit. The arrays were scanned using the Affymetrix GeneChip^®^ Scanner 3000 7G, and the signal intensity of probe hybridization was processed using the Affymetrix^®^ GeneChip^®^ Command Console^®^ (AGCC) Software.

Statistical analysis of microarray data were performed using R software (version 3.1)^51^ and the affy^52^, affycoretools^53^, statmod and Bioconductor Limma^54^ packages. Raw data was initially assessed and normalized using the robust multichip analysis (RMA) algorithm. Differential gene expression between groups was then determined by fitting a linear model to the data using lmFit with subsequent comparisons made using the makeContrasts function. Transcripts with a q-value^55^ of less than 0.05 were classed as significantly differentially regulated. Further analysis was performed using Expressionist from GeneData.

### RNAi knockdown

RNAi knockdown was performed by feeding using the *C. elegans* RNAi v1.1 Feeding Library from Open Biosystems which is derived from the *C. elegans* ORFeome Library. This is in the form of glycerol stocks of *E. coli* with each strain expressing dsRNA against one *C. elegans* open reading frame. Strains were grown up in LB containing ampicillin and were used to seed 5 cm and 24 well NGM agar plates containing ampicillin and IPTG. These plates were then induced by being placed at 37 °C overnight before use.

Worms were bleached to recover isolated eggs^50^. These eggs were added to the 5 cm RNAi plates and placed at 15 °C for four days to reach L4. After four days AI was added to the 24 well RNAi plates as described for the egg viability assay. The L4 were then picked onto the 24 well AI containing RNAi plates for the egg viability assay.

## Additional Information

**How to cite this article**: Harlow, P. H. *et al*. The nematode *Caenorhabditis elegans* as a tool to predict chemical activity on mammalian development and identify mechanisms influencing toxicological outcome. *Sci. Rep*. **6**, 22965; doi: 10.1038/srep22965 (2016).

## Supplementary Material

Supplementary Information

## Figures and Tables

**Table 1:** The egg viability assay in *C. elegans* predicts developmental toxicity in mammals.

		Developmentalactivity in mammals		Positivepredictivity	Negativepredictivity
		active	inactive		
**Developmentalactivity in*C. elegans***	**active**	17	2	89%	
	**inactive**	40	13		25%
**Sensitivity**		30%			
**Specificity**			87%		

The number of developmentally active and inactive compounds in *C. elegans* and mammals is recorded. Positive predictivity is the percentage of compounds found to be developmentally active in *C. elegans* that are also developmentally active in mammals. Negative predictivity is the inverse: the percentage of compounds found not to be developmentally active in *C. elegans* that are also not developmentally active in mammals. Sensitivity is the percentage of compounds that are developmentally active in mammals that were detected by the assay as developmentally active in *C. elegans*. Specificity is the percentage of compounds that are not developmentally active in mammals that are not detected by the assay as developmentally active in *C. elegans*. Developmental activity in *C. elegans* was assayed as egg viability following maternal exposure to test compounds as described in Methods. Mammalian developmental activity was calculated from the ToxCast database^24^ as described in Results.

**Table 2:** Results of the RNAi screen of cytochrome P450 genes in *C. elegans*.

Average %unhatched	Compound 7		Compound 6		Solvent	
	50 μg/ml	5 μg/ml	0.5 μg/ml	0.05 μg/ml	1	2
*cyp-13A1,2,3*	26	2	1	1	2	2
*cyp-13A6,7*	15	5	7	2	1	3
*cyp-13B1,2*	10	4	2	3	1	4
control 1	30	5	26	4	5	12
						
*cyp-14A1,2*	40	3	13	4	3	1
*cyp-14A4,5*	13	2	13	3	3	3
*cyp-33C1,2,4*	16	3	18	2	1	8
control 2	22	9	8	2	5	8
						
*cyp-33C3,6,7*	16	2	5	1	2	1
*cyp-33C8,9,11*	9	2	3	1	2	3
*cyp-33B1,D1,D3*	11	3	3	0	1	2
control 3	16	4	10	1	2	2
						
*cyp-29A2, 33E1,3*	11	2	3	1	2	2
*cyp-34A1,2,3*	9	1	7	2	2	2
*cyp-34A4,5,6*	18	3	2	1	2	1
control 4	17	6	7	5	4	6
						
*cyp-34A7,8*	5	2	1	1	2	2
*cyp-34A9,10,36A1*	15	1	3	1	1	2
*cyp-35B1,3,D1*	16	1	2	1	2	1
control 5	10	1	1	1	1	2
						
*cyp-35A2,A5,C1*	28	2	37	7	5	5
*cyp-35A3,4*	79	3	89	10	1	1
*cyp-37A1,B1,43A1*	0	3	3	2	2	5
control 6	9	1	6	0	4	3
						
*cyp-13A4,5,11*	26	2	9	2	2	13
*cyp-25A1,2,33E3*	11	1	1	2	4	1
*cyp-25A4,5*	13	2	12	0	2	2
control 7	19	5	10	1	0	0

Egg viability (n = 4–5) was recorded after exposure to compounds **6** & **7** in animals in which 1-3 cytochrome P450 genes had been knocked down by RNAi (see Methods). Numbers are the percentage of eggs laid that did not hatch. Combined RNAi treatments are in rows. For example, *cyp-25A1,2,33E3* indicates that 3 *E. coli* strains expressing an RNAi construct targeting either *cyp-25A1*, *cyp-25A2* and *cyp-33E3* were mixed and presented to test animals. Compound treatments are in columns. Blank rows separate individual experiments. All statistical comparisons are made within each experiment. Doses shown do not cause maternal lethality but are either side of the maternal toxicity NOEL.
