# Supplementary material for: The nematode Caenorhabditis elegans as a tool to predict chemical activity on mammalian development and identify mechanisms influencing toxicological outcome
Source: Sci Rep. 2016 Mar 18;6:22965. doi: 10.1038/srep22965 (PMC4796825; doi:10.1038/srep22965)
Supplement: Supplementary Information [file srep22965-s1.pdf]

The nematode *Caenorhabditis elegans* as a tool to predict chemical activity on mammalian development and identify mechanisms influencing toxicological outcome.

Philippa H. Harlow[1], Simon J. Perry[1], Stephanie Widdison[2], Shannon Daniels[3], Eddie Bondo[3], Clemens Lamberth[4], Richard Currie[1], and Anthony J. Flemming[1]*,

[1] Syngenta Ltd., Jealott's Hill Research Station, Bracknell, Berkshire, RG42 6EY, UK

[2] General Bioinformatics, Jealott's Hill Research Station, Bracknell, Berkshire, RG42 6EY, UK

[3] Syngenta, 3054 East Cornwallis Road, Research Triangle Park, NC 27709-2257, USA

[4] Syngenta Crop Protection AG, Chemical Research, Schaffhauserstrasse 101, 4332 Stein, Switzerland

*anthony.flemming@syngenta.com

Supplementary information

Developmentally toxic compounds		*Negative controls*	
Chlorophacinone		Methomyl	
Flusilazole		Tribufos	***
Oxamyl		Ethoprop	
Diniconazole	***	Benfluralin	
Hexaconazole		Dithiopyr	
Tralkoxydim		Tebufenozide	
Fluazifop-P-butyl	***	Fipronil	
Atrazine		Cyclanilide	
Bis(tributyltin)oxide		Zoxamide	
Triadimenol		Bifenazate	
Monosodium methane arsenate		Methoxyfenozide	
Ethylenebisdithiocarbamate, disodium	***	Foramsulfuron	
Thiacloprid		Cypermethrin	
Spiroxamine		Triclosan	***
Acibenzolar-S-Methyl		Fosthiazate	
Flumioxazin			
Cymoxanil			
Isoxaflutole			
Sulfluramid	***		
Dicrotophos			
Cyhexatin			
Diquat dibromide			
Thiophanate-methyl			
Cyproconazole			
3-(3,5-Dichlorophenyl)-1,5-dimethyl-3-azabicyclo(3.1.0)hexane-2,4-dione	***		
Carbendazim	***		
Pymetrozine			
Molinate			
Paclobutrazol			
Fluazinam	***		
S-Bioallethrin			
Benomyl	***		
Iodosulfuron-methyl-sodium			
Mesotrione			
Fludioxonil			
Prodiamine			
Butralin	***		
Mecoprop-P	***		
Norflurazon	***		
Triadimefon			
Topramezone			
Acetic acid, {(5-chloro-8-quinolinyl)oxy}-, 1-methylhexyl ester	***		
Trifloxysulfuron-sodium			
Tepraloxydim			
MCPP Acid	***		
Acetochlor	***		
Iprodione	***		
1,2-Butanediol			
Fluoxastrobin			
Primisulfuron-methyl			
Quintozene			
3-Aminotriazole	***		
Cyazofamid			
Spirodiclofen	***		
Sethoxydim			
Imazapic			
Fluthiacet-methyl			

Supplementary Table 1. The full list of compounds from the ToxCast database tested in the egg hatching assay with the results (*** indicates p<0.001 decrease in egg viability compared to control).

Compound	Conclusion of preliminary study on the rat.
**2**	The dose level of 75 mg/kg/day was concluded to be adverse to the developing foetus resulting in death and abnormality
**3**	Substantial effects on pregnancy and foetal development were seen at a dose level of 30 mg/kg/day.
**5**	No major or minor fetal malformations / abnormalities were observed at 200 & 400 mg/kg/day. At the limit dose of 1000 mg/kg/day, a single fetus was malformed. This could potentially be background variation.
**6**	Administration of the high dose of 300 mg/kg/day resulted in a slight, but not excessive, reduction in overall bodyweight gain for the treatment period and slight increases in pre- and post-implantation loss. Intra-animal variation was apparent and, in view of the small group sizes on this study, a relationship to treatment with **6** was considered to be unclear.
**10**	The major foetal abnormalities apparent at 200 and 500 mg/kg/day were considered to be related to maternal treatment with **10**.
**15**	No evidence of maternal toxicity, but major malformations of fetuses were observed at 400 and 1000 mg/kg/day along with minor abnormalities across all doses. The findings from the study, including lower survival of fetuses, the higher number of malformations and the increase in fetuses with visceral variations compared to controls, lead to the conclusion that disruption to normal development was apparent following treatment with this compound.

Supplementary Table 2. Conclusions of preliminary developmental toxicity studies in the rat. Preliminary to OECD guideline developmental toxicity studies were performed at a specialist contract research organization in the UK in accordance with all relevant legislation, ethical approvals and experimental best practice. Groups of 6-10 time mated female rats received AI by oral gavage daily for 19-21 days starting at day 5-6 of pregnancy. The vehicle and control was 0.5 % aqueous carboxymethycellulose. A constant dose volume of 10 mL/kg was used. All animals were examined for effects on general condition, body weight and food consumption and the progress and outcome of pregnancy. Blood samples were collected twice during gestation to demonstrate exposure to the test article.

Gene name	**3**	**4**	**6**	**7**	**16**	**17**
cyp-13A1	0.73	0.76	0.81	0.73	0.73	0.68
cyp-13A10	1.41	1.77	1.11	3.89	1.72	5.45
cyp-13A11	1.00	1.03	1.40	9.70	1.22	1.24
cyp-13A2	1.22	1.27	1.16	1.21	1.13	1.56
cyp-13A5	0.72	0.71	0.84	2.24	0.60	0.58
cyp-13A6	1.05	1.12	1.19	2.18	1.03	1.46
cyp-13A7	0.85	0.87	1.00	2.55	0.98	0.91
cyp-13A8	1.02	1.19	0.98	2.94	1.00	0.96
cyp-13B1	0.82	1.13	1.04	1.22	1.48	1.08
cyp-13B2	1.32	1.11	0.95	1.41	1.14	1.11
cyp-14A2	1.21	2.64	1.23	1.09	1.42	1.66
cyp-14A3	1.27	5.06	1.03	1.15	1.28	1.55
cyp-14A5	3.36	3.11	1.51	2.40	3.22	4.00
cyp-29A3	1.44	1.74	1.29	1.31	1.67	1.66
cyp-32A1	1.40	1.62	1.34	1.42	2.05	1.91
cyp-32B1	0.53	0.68	1.07	0.28	0.81	0.46
cyp-33B1	1.07	0.87	0.75	0.36	0.76	1.15
cyp-33C2	1.05	1.14	1.02	1.19	1.10	3.10
cyp-33C4	0.94	1.05	0.89	1.01	0.92	1.59
cyp-33C9	0.79	0.97	1.08	0.66	0.99	0.87
cyp-33D3	1.01	1.10	1.11	2.13	1.09	1.10
cyp-33E1	0.67	0.83	0.82	0.52	0.87	0.71
cyp-33E2	0.93	1.08	1.00	1.03	1.30	2.06
cyp-33E3	1.04	1.09	1.12	1.08	1.26	1.32
cyp-34A6	0.92	1.00	0.98	0.86	0.89	0.81
cyp-34A7	1.08	1.15	1.01	1.51	0.97	1.15
cyp-34A8	1.18	1.37	0.98	0.91	1.20	1.53
cyp-34A9	0.69	0.82	0.95	0.57	0.89	0.92
cyp-35A1	1.02	1.01	1.07	1.52	1.30	1.56
cyp-35A2	0.72	0.66	2.48	4.30	1.75	2.39
cyp-35A3	1.00	0.83	26.12	111.47	5.82	31.77
cyp-35A4	1.01	1.04	11.81	52.40	1.64	6.20
cyp-35A5	1.09	1.13	6.05	14.34	2.38	3.31
cyp-35B1	0.61	0.99	1.60	0.59	1.12	0.92
cyp-35B2	1.04	1.88	2.25	2.85	1.58	1.97
cyp-35C1	0.94	0.95	21.80	48.82	6.89	10.38
cyp-35C1	0.96	1.16	18.28	34.71	7.49	10.51
cyp-37A1	0.67	0.77	1.19	0.50	1.06	0.68
cyp-37B1	0.53	0.54	0.88	0.26	0.71	0.53

Supplementary Table 3. Cytochrome P450 genes with altered expression following exposure to compounds 3, 4, 6, 7, 16, 17. Numbers indicate fold change compared to control. Highlighted genes are differently expressed compared to control (adjusted P < 0.05). Blue shading indicates upregulation. Red shading indicates downregulation.

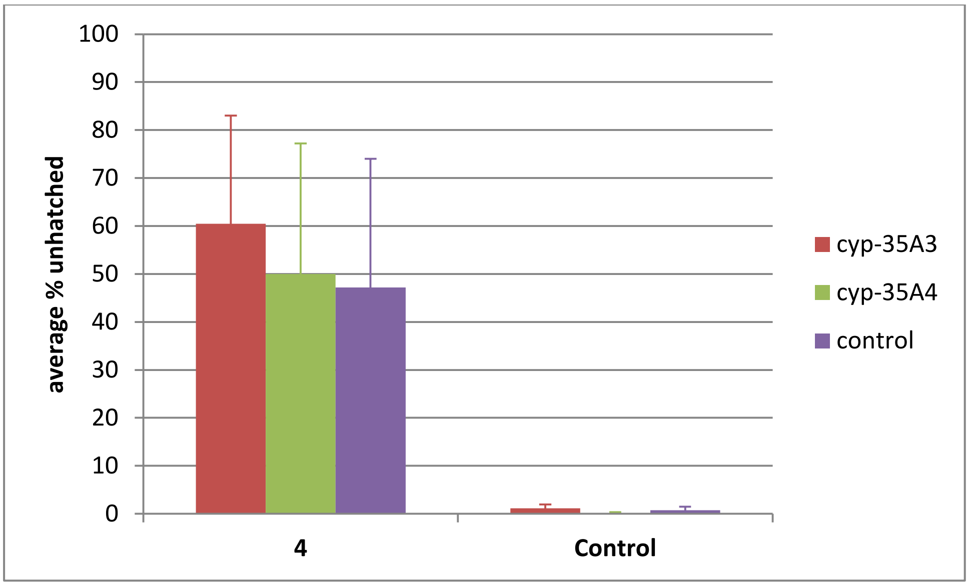

Supplementary Figure 1. ***C. elegans*** **treated with** ***cyp-35A3 RNAi*** **and** ***cyp-35A4 RNAi*** **separately are not more sensitive to the developmentally toxic effects of compound 4.** The dose was 0.05µg/ml compound **4.** This dose induces developmental toxicity but not maternal toxicity in *C. elegans* N2 .The columns show mean ± s.e. of n=4. Purple column shows compound no RNAi control. Control on x axis is RNAi no compound control.
